# Immunoglobulin M in Health and Diseases: How Far Have We Come and What Next?

**DOI:** 10.3389/fimmu.2020.595535

**Published:** 2020-10-30

**Authors:** Katelyn Jones, Anca F. Savulescu, Frank Brombacher, Sabelo Hadebe

**Affiliations:** ^1^ Division of Immunology, Department of Pathology, Faculty of Health Sciences, University of Cape Town, Cape Town, South Africa; ^2^ Division of Chemical, Systems & Synthetic Biology, Faculty of Health Sciences, Institute of Infectious Disease & Molecular Medicine, University of Cape Town, Cape Town, South Africa; ^3^ Division of Immunology, Health Science Faculty, International Centre for Genetic Engineering and Biotechnology (ICGEB) and Institute of Infectious Diseases and Molecular Medicine (IDM), University of Cape Town, Cape Town, South Africa; ^4^ Wellcome Centre for Infectious Diseases Research in Africa (CIDRI-Africa), Faculty of Health Sciences, Institute of Infectious Diseases and Molecular Medicine (IDM), University of Cape Town, Cape Town, South Africa

**Keywords:** immunoglobulin M (IgM), B cell development, short-lived plasma cell (SLPC), long-lived plasma cell (LLPC), memory B cell (MBC)

## Abstract

B lymphocytes are important in secreting antibodies that protect against invading pathogens such as viruses, bacteria, parasites, and also in mediating pathogenesis of allergic diseases and autoimmunity. B lymphocytes develop in the bone marrow and contain heavy and light chains, which upon ligation form an immunoglobulin M (IgM) B cell receptor (BCR) expressed on the surface of naïve immature B cells. Naïve B cells expressing either IgM or IgD isotypes are thought to play interchangeable functions in antibody responses to T cell-dependent and T cell-independent antigens. IgM short-lived plasma cells (SLPCs) and antigen-specific IgM memory B cells (MBCs-M) are critical in the first few days of infection, as well as long-term memory induced by vaccination, respectively. At mucosal surfaces, IgM is thought to play a critical part in promoting mucosal tolerance and shaping microbiota together with IgA. In this review, we explore how IgM structure and BCR signaling shapes B cell development, self and non-self-antigen-specific antibody responses, responses to infectious (such as viruses, parasites, and fungal) and non-communicable diseases (such as autoimmunity and allergic asthma). We also explore how metabolism could influence other B cell functions such as mucosal tolerance and class switching. Finally, we discuss some of the outstanding critical research questions in both experimental and clinical settings targeting IgM.

## Introduction

IgM is the first antibody isotype expressed during B cell development and the first humoral antibody responder, conserved across all species from Zebrafish to humans (1). In cartilaginous and bony fish, IgM has been found to have crucial functions in host defense and tolerance (2). IgM can be divided into natural and antigen-induced IgM and can either be membrane bound IgM-type BCR or secreted IgM (3, 4). Natural IgM plays multiple roles in homeostasis including scavenging, B cell tonic signals for B cell survival, lymphoid tissue architecture, and prevention of autoimmune diseases (5, 6). IgM is involved in clearance of debris, particles (below 2 μM) and apoptotic cells through antibody dependent opsonization and phagocytosis by macrophages (7, 8). At mucosal sites both natural and antigen-induced IgM play a role in shaping healthy microbiota and their repertoire, although limited, is also shaped by microbiota (9, 10). Secreted IgM antigen-complexes can connect signals *via* unique and shared receptors, suggest a more pleotropic role in homeostasis and disease states (11, 12).

Since the discovery of individuals with selective IgM deficiency, a lot has been learnt about IgM in various human diseases including autoimmune and infectious diseases (13, 14). Genetically conditioned mice which lack secreted or membrane bound IgM have underscored the importance of IgM in many infectious diseases. In this review, we highlight what is currently known about the role of IgM in B1 and B2 cell development, memory, and plasma cell generation, in and outside GCs. Lastly, we discuss experimental models using IgM-deficient mice and corroborating phenotypes observed in humans with selective IgM deficiency.

## B Cell Development

### Naturally Occurring Immunoglobulin M B Cells (B1)

**Figure 1:**
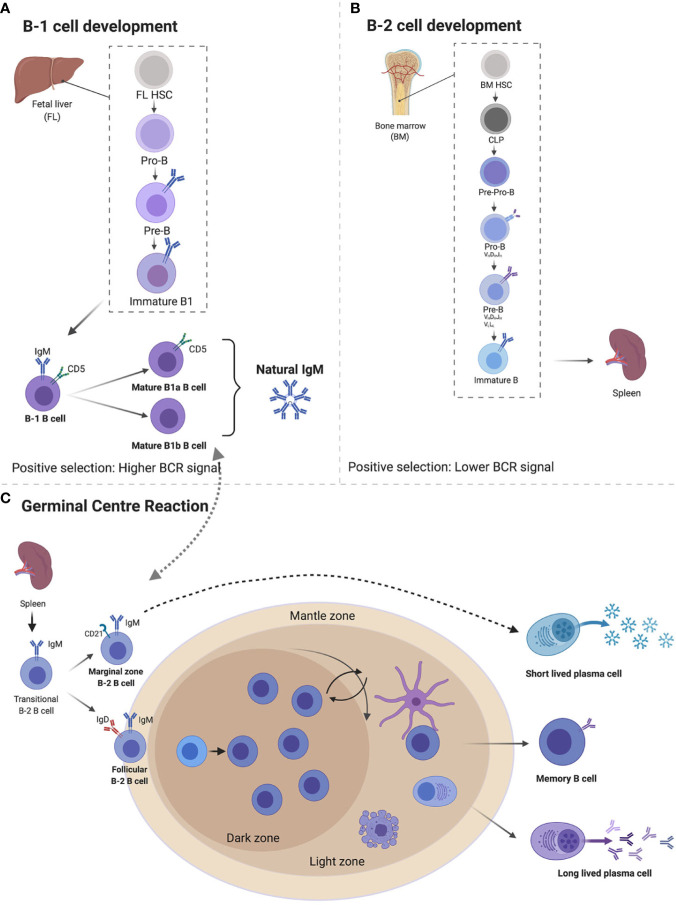
Immunoglobulin M (IgM) developmental pathways through B1 and B2 B cells from fetal liver (FL) and bone marrow (BM). B1 cells develop FL where they go through pro-B cell, pre-B cell, immature B cell, and naïve B cells expressing IgM and CD5 which differentiates B1a and B1b cells, both capable of secreting natural IgM **(A)**. B2 cells develop from BM’s common lymphoid progenitor to become immature B cells that migrate to splenic B cells secreting IgM. Expression of IgD differentiates marginal zones *vs.* follicular B cells **(B)**. Follicular B cells upon antigen stimulation can either undergo germinal center maturation creating long-lived plasma cells, memory B cells, class switch, or remain unswitched short-lived plasma cells **(C)**. Created with BioRender.com.

B1 cells develop in the yolk sac on embryonic day 9, before birth from a functional hematopoietic stem cell subset termed the common lymphoid progenitor, in the fetal liver and seed the peritoneal and pleural cavities (15–21). B1 cells are thought to be the main source of naturally occurring IgM, although there is controversy on the main contributing organ, with some studies suggesting bone marrow (BM) and spleen B1 cells as important sources (22). B1 cells are thought to lack specificity and affinity maturation similar to innate immune receptors and are referred to as innate-like B cells or unconventional (4, 16). The concept of non-specificity is somewhat nullified by the fact that B1 cells are polyreactive—they recognize polysaccharides found on the cell wall surfaces of a wide array of pathogens, but with exquisite specificity (23, 24). This specificity allows them to confer protection against pathogens bearing similar epitopes (discussed later). Furthermore, B1 cells are self-reactive and develop normally in the absence of foreign antigen stimulation, suggesting that their development is self-regulated *via* a mechanism of binding to glycosylated and oxidized mammalian molecules to prevent self-recognition (15, 20, 25). B cell receptor is intricately regulated by CD5 (Ly1) which enables self-antigen recognition and some level of specificity (**Figure 1A**) (20, 26).

The majority of B1 cells are found in the peritoneal cavity where they are self-renewing and undergo maintenance with the help from resident macrophages that secrete CXCL13 (27). Other sites such as spleen, lymph node, bone marrow, pericardium, and mucosal associated lymphoid tissue account for as little as 1% of B1 total pool (11, 22, 28, 29). The phenotype of B1 cells varies depending on the compartment, with splenic B1 cells and peritoneal B1 cells displaying different antibody repertoire, gene expression, and secretion of IgM (16). In the peritoneal cavity, B1 cells can be identified by surface expression of CD19^hi^, B220^low^, CD43^+^ CD5^+^/CD5^low/−^, CD23^low^, CD11b^+^, whereas in other tissues, where they migrate after injury, they lose CD11b expression as they become plasma cells, making it difficult to differentiate them with B2 cells in these tissues (16, 26). B1 cells are divided into B1a (CD5^+^) and B1b (CD5^−^), with B1a cells accounting for the majority of the B1 cell population (16, 20, 25, 30, 31). While B1b cells can potentially develop from bone marrow progenitors, B1a cells cannot (30, 32). Both B1a and B1b cells display similar surface markers with the exception of CD5, which regulates B1a cell autoreactivity (16, 26).

### Bone Marrow Derived Immunoglobulin M B Cells (B2)

Conventional B cells (B2) are derived from bone marrow after birth from a common lymphoid progenitor (CLP) and their commitment to B cell lineage is dependent on the BM microenvironment (25) (**Figure 1B**). B cell lymphopoiesis is a rather complex process. Here, we give a brief summary mainly to illustrate how naïve B cells exiting the BM expressing surface IgM reach peripheral tissues. For more detailed reviews on this topic, we refer the reader to a number of review articles (33–35). The subsequent stages are important in a B cell’s development and they introduce diversity into the antibody’s repertoire (36). The first stage is a pre-pro B cell, where initial diversification of the D and J segments occurs, followed by the pro-B cell where recombination of the V region to the previously rearranged D-J is completed (33–36). Interleukin 7 (IL-7) from stroma and IL-7Rα signaling on developing B cells play both positive and negative regulatory roles in B cell development, allowing proliferation and pro-survival signals, as well as switching off recombination for next stage of development (34, 37). Following a successful V-D-J rearrangement in the pro-B cell, expression of the Igμ heavy chain (μHC) in the pre-B cell stage occurs. The V and J segments of the two germline-encoded surrogate light chain (VpreB and lamda5), combine with an existing Igμ heavy chain (33). This is then followed by association with signaling subunits Igα and Igβ and assembly, resulting in surface expression of the pre-BCR (34–36). The pre-B cells are large and motile and act in positive selection to select against autoreactivity, making the pre-B cell stage a tolerance checkpoint (33, 38, 39). Recombination activating genes 1/2 (RAG1/RAG2) are key in the progenitor B cell development and allow genetic recombination rearrangement (40). The final transition of these large proliferating pre-B cells before they exit the BM occurs as they move away from the IL-7 rich stromal region, downregulate IL-7Rα and induce the expression of the IRF4 transcription factor (37, 41). IRF4 induces transcription of CXCR4, which in turn inhibits proliferation and cell cycle exit, as well as inducing reduction in size of the pre-B cell. RAG1/RAG2 allow for a final recombination of the V and J regions of the light chain (Igκ and IgL) in the CXCL12-CXCR4 rich environment and development of the immature B cell (25) (**Figure 1B**). The immature B cells leave the BM *via* vascular sinuses and migrate to the peripheral tissues such as the spleen and lymph nodes where they complete their final maturation (38).

### Peripheral B Cell Maturation and Production of Immunoglobulin M by B2 Cells

The regulation of B cell development is mediated by the BCR when transitioning from an immature to a mature B cell (25). An immature transitional B cell undergoes several splicing events and primary variable diversity joining of Cμ and Cδ transcripts (42, 43). This leads to a naïve B cell co-expressing both IgM and IgD BCRs isotypes on the surface, with identical specificities (43, 44). These naïve B cells still display a certain level of self-reactivity and are further pruned through clonal deletion and anergy, where they can become unresponsive to self-antigen stimulation, thus preventing autoimmunity (25, 42, 44–46). Transitional B cells localize in secondary lymphoid tissues such as the spleen or lymph nodes, where they spatially sub-localize in follicular regions for easy access to both sampling of antigens and a local area rich with B cell survival factors, such as BAFF (25, 46). The naïve B cells are attracted to follicular areas by CXCL13 chemokines and once they encounter antigens, they upregulate CCR7, which enables them to sense CCL21- and CCL19-rich T cell zone areas (46). At this stage, B cells seek T cell help for a cognate antigen, which further stimulates their survival, proliferation, and antibody secretion function (47). In order for antigen primed naïve B cells to have access to highly competitive T cell help, they need to undergo several rounds of high affinity maturation to create clones that are likely to survive longer and possibly create long term memory (**Figure 1C**). These processes take place in the germinal centers (GCs), which are secondary B cell follicle areas (47, 48). Naïve B cells that do not to take part in the GC reaction are pushed to the B cell mantle zone, where they divide and form short-lived plasmablasts, which eventually produce low affinity short-lived IgM plasma cells. The GC [identified by GL7 and Fas (CD95) expression] is a highly proliferative area, divided into the light zone (LZ) and dark zone (DZ) (48). The LZ contains follicular dendritic cells (FDCs), where selection of BCRs takes place (48). The B cells receive the antigen from FDCs, present it to T follicular helper (Tfh) cells; if the mutation confers an advantage, the specific cell will be selected (48, 49). The DZ is the area in which where somatic hypermutation (SHM) takes place and it appears dark, due to the densely packed B cells that proliferate (**Figure 1C**) (47, 48). In the DZ, *Aicda*, a gene that encodes for activation-induced deaminase (AID) is highly expressed. AID deaminates cytidine residues in the VDJ and switch regions of the Ig gene, leading to SHM and class switch (47, 50). During SHM, AID catalyzes the deamination of C to U, to activate error prone repair pathways to induce mutations (51, 52).

Class switching, which occurs in the GC and occasionally in extrafollicular sites (47, 53), involves the replacement of the H-chain C-region for another Ig gene, for example mμ (IgM) for gamma (IgG) (36). The constant region (Fc) of the BCR changes, while the variable side (Fab) remains constant, therefore the antigen specificity prevails. However, various signaling cascades and immune responses occur, based on the class of Ig that is present. Within the GC population, IgG/IgM cells ratio remains constant, indicating a dynamic steady state between class switched and non-class switched cells (53). The process of antibody class switching is evolutionary conserved across species and is found as early in evolution as cartilaginous sharks and *Xenopus* (54). In the South African clawed frog (*Xenopus laevis*), IgM shows limited antibody repertoire and reduced affinity despite reasonable mutation rates compared to mammals (54). The limiting factors for IgM affinity in clawed frogs and sharks appear to be a lack of germinal center (GC) B cell compartment, as well as reduced AID-dependent somatic hypermutations that are found in mammals (54, 55). This limited mutation rate is at least partially evolutionary conserved, as it is observed in certain long-lived memory IgM B cells or low affinity memory B cells generated outside GCs in humans (56, 57).

## Structure of Immunoglobulin M and Antigen Recognition

**Figure 2:**
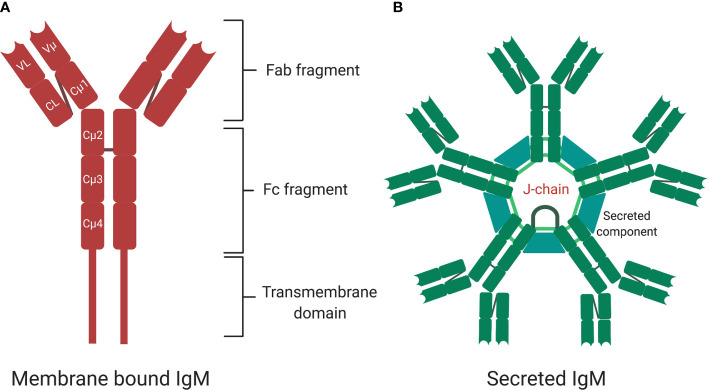
The structure of membrane bound and secreted immunoglobulin M (sIgM). **(A)** A monomer structure of immunoglobulin M (IgM) contains Fab fragments, Fc fragment, and transmembrane signaling tail that attaches to Fc receptors on the surface of B cells. A monomer is made up of two heavy chains and two light chains. **(B)** A pentamer structure is the most naturally occurring form with five monomers held together by a J-chain. Secretary component regulates surface availability and secretion of the pentamer. Created with BioRender.com.

IgM exists in two forms—membrane bound (mIgM) and secreted (sIgM), with sIgM being further divided into natural and antigen induced IgM (**Figure 2**) (5, 6). IgM can exist in various structural forms including a monomer, a hexamer, and a pentamer, the latter weighing over 1,000 kDa (6, 58). Pentameric assembly of sIgM is the most naturally occurring form, with monomers held together by a 15-kDa protein J-chain that bridges disulfide bonds *via* a C-terminal extension of the heavy-chain (**Figure 2B**) (59–62). IgM typically displays low binding affinity to antigens, however, the multivalent antigen-binding sites in the pentameric structure of sIgM and its multivalent antigen-binding sites lead to high avidity for antigens, ensuring efficient elimination of pathogens (63, 64). Similar to other antibody structures, IgM BCR is composed of two homodimeric heavy chains, each bearing a light chain linked *via* disulfide bonds (65, 66). The μ region of the heavy chain folds into four domains, with the constant µ domain 4 (Cµ4) allowing anchoring of the membrane bound IgM to the surface of the B cell and activation of complement (**Figure 2A**) (67, 68). The membrane bound IgM BCR is essential for B cell development and activation, *via* the phosphoinositide 3-kinase pathway (**Figure 2A**) (69–71). The role of hexameric IgM structure is currently unclear, but it is thought to exist due to defects in the μ chain or J-chain regions in pentameric IgM (72). Secretion of IgM is regulated by the secretary component (SC) and J-chain (**Figure 2A**), which regulate surface availability of IgM and premature release through preventing protease cleavage, particularly in mucosal sites where there is richness in microbes that often use these mechanisms to evade host recognition (61). Apart from regulation by SC and J-chain, sIgM is also post-translationally modified through N-glycosylation and sialylation (60, 73). Most of the N-glycosylation sites are in the μ chain and with one site in the J-chain and mutations in these sites lead to accumulation of IgM on the cell surface and reduced secretion (73, 74).

**Figure 3:**
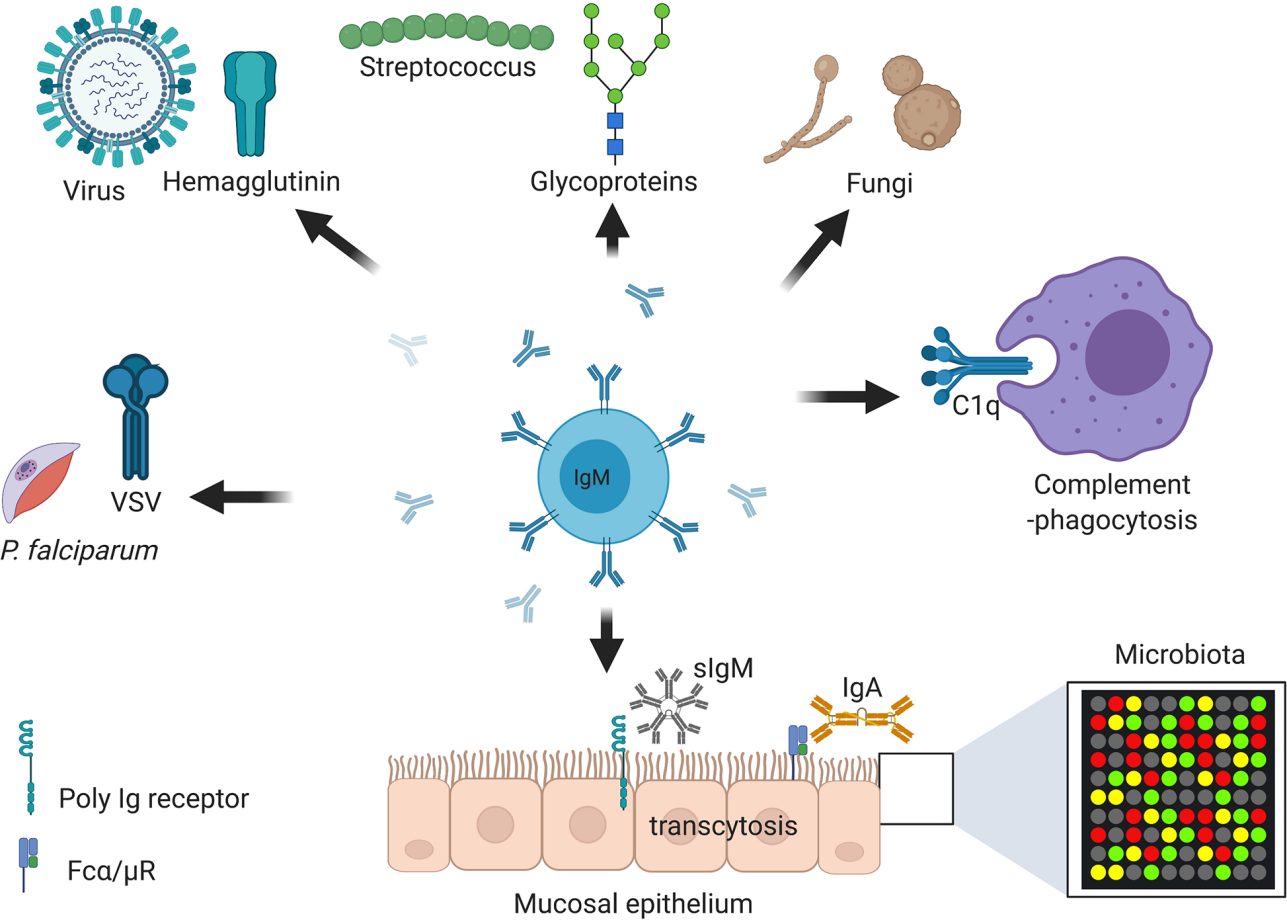
Immunoglobulin M (IgM) is central at steady stage and against infections and non-communicable diseases. Secretory IgM is important at mucosal surfaces in maintenance of healthy microbiota together with secreted IgA. Secretory IgM together with IgM B cell receptor are important in initiation protective immunity against various respiratory pathogens including species of fungi, viruses, and bacteria. Secreted IgM is essential in parasitic infections including those causing malaria and sleeping sickness. Secreted IgM play an important part in cancers diagnosis and auto-immunity diseases such as systemic lupus erythematosus (SLE) and rheumatoid arthritis (RA). Secreted IgM has high affinity for C1q, a complement component that allows degradation of antibody coated pathogens and apoptotic debris. Created with BioRender.com.

Upon binding to surface-exposed antigens *via* antibody binding region (Fab), pentameric IgM complexes undergo conformational changes (68) followed by interaction of the antibody-antigen complex with B cells receptors *via* binding of the constant (Fc) domain. IgM can bind to several cell surface receptors including complement receptor CR2 and CR3, polymeric Ig receptor (pIgR), Fcα/μR and FcμR on B-cells, epithelium cells, and antigen presenting cells (75–77). FcμR specifically binds sIgM in mice and exclusively so in human (77). Mice deficient in FcμR expression exhibit spontaneous GC formation, long-lived plasma cell development and memory B cell formation (76, 78). The polymeric Ig receptor is expressed at the basal membrane of mucosal epithelium and exocrine glands and binds to sIgM and sIgA to mediate transcytosis of these antibodies from lamina propria or ileum to apical mucosal sites where they bind to microbiota (**Figure 3**) (79, 80). Fcα/μ receptor (Fcα/μR) is expressed in non-hematopoietic cells and by marginal zone B-2 cells (81, 82). Binding of IgM-antigen complexes to the Fcα/μR has been shown to mediate endocytosis and pro-inflammatory cytokine production (81, 82).

## Immunoglobulin M Antibody Responses to T Cell-Dependent and -Independent Antigens

Class switching, which occurs in the germinal centers (GCs) and occasionally in extrafollicular sites (36, 47, 53), involves intrachromosomal rearrangement of the Ig heavy chain C-region from Cμ (IgM)/Cδ (IgD) with Cγ1 (IgG) without altering specificity to immunizing antigen (36, 52). This class switching is thought to occur sequentially in GCs (83–87). However, direct class switching from Cμ to Cε or Cα has been observed, particularly in extrafollicular GCs where it is partly thought to be driven by lack of B cell maturity or low activation threshold (83, 88, 89). Class switch recombination is initiated by AID, which targets intronic switch (S) regions, causing DNA breaks and recombination of the heavy chain VDJ segments with other constant regions (50, 51, 84, 87). It is widely thought that memory B cells are generated from antigen T-dependent interactions that take place in the GC and that the majority of first wave plasma cells are of IgM isotype, short-lived, display high avidity and are T-independent (53). Over the last decade, increasing evidence suggests that memory B cells of IgM isotype exist and that these cells can secrete long-lived plasma cells (LLPCs) when stimulated by a cognate antigen (57). The nature of the generation of IgM memory B cells generation is rather complex, as it seems to depend on the tissue of origin (local events), GCs or extrafollicular GCs pathway and SHM rate of integrative genomics viewer (IgV) region of B cell receptor (57, 88, 90–93).

### Antigen-Specific Immunoglobulin M Short-Lived Plasma Cells

Short-lived plasma cells (SLPCs) of IgM producing antibodies are typically found in the spleen on the periphery of B cell follicles, displaying little to no SHM (**Box 1A**) (92). Long-lived plasma cells on the other hand, show some degree of high affinity, suggestive of having gone through GCs, and, can be found in the BM (**Box 1B**) (91). Short-lived plasma cells’ differentiation is governed by the B-cell lymphoma 6 (BCL6) and PR domain containing 1 (PRDM1)/BLIMP-1 transcription factor (94, 95). BCL6 favors GC entry, whereas BLIMP-1 represses BCL6 and favors antibody secreting cells (ASCs). Interactions showing poor strength between BCR and antigen favor higher avidity, tend to be generated in extrafollicles and do not enter GCs resulting in SLPCs (94, 96). These SLPC release the early wave of antibodies post-antigen exposure and provide the initial protective response prior to emergence of high affinity antibodies (94, 96). Additional evidence suggests a key role for the glycolysis pathway in this T-independent SLPC production (97). This process involves mechanistic target of mTOR activating transmembrane activator and CAML interactor (TACI) *via* MyD88, to induce MZ B cell proliferation and genetic recombination, allowing non-GC class switching (97). LLPCs of IgM isotype were only described recently and differ from IgG LLPCs, as they develop independently of GCs (57). This population persists in the spleen, unlike IgG BM-residing LLPCs and undergo SHM (some outside of the GC), in an AID-dependent and BLC6-independent manner (57, 98, 99). The mutations that occur are not typically in the complementarity determining region 3 (CDR) and are therefore not thought to be selected for by antigen affinity (57). The IgM LLPCs are capable of conferring protection against viral and bacterial infections *in vitro* and *in vivo*, independently of IgG LLPCs, memory B cells, and T cell help (32, 57).

### Antigen-Specific Immunoglobulin M Memory B Cells

Antigen specific IgM memory B cells (MBCs-M) form a subset of memory B cells that secrete IgM in the spleen, surprisingly also in germ-free mice, albeit with reduced diversity (**Box 1C**) (56, 100). MBCs were initially described as being IgG or IgA isotypes and expressing high levels of CD73, CD80, and PD-L2. However, it is now accepted that an MBC-M population exists from an early GC reaction and lacks classical MBCs surface molecules (100–102). MBCs-M show poor affinity compared to MBCs-G and contain less IgV mutations, however, their half-life is significantly longer (99). The mechanisms by which MBC-Ms survive longer and are more persistent remain largely unclear. BCR avidity and usage (CDR3 *vs.* non-CDR3) and mouse genotype rather than antigen are thought to be key in the persistence (95). Although, MBC-M are in many ways similar to naïve B cells, they show different dynamics in GC entry and ASCs production (103). Compared to MBCs-M, naïve B cells express considerably higher levels of Krüppel-like factor (KLF) 4, KLF9, and promyelocytic leukemia zinc finger (PLZF), transcription factors associated with quiescence (104). It is likely that these factors repress genes associated with survival and cell cycle, allowing significantly faster turnaround in ASC production and if needed, generation of class switched plasma cells (94). An additional important aspect that has emerged as key in class switching and plasma cell generation is metabolism (105). A recent study showed that naïve follicular B cells entering GCs prefer fatty acid oxidation over glycolysis as an energy source (106). It is likely that differential metabolite needs may have further upstream implications, particularly in MBCs-M function, be it ASC production or re-entry into GCs for further SHM.

MBCs-M acquire high affinity BCRs through SHM upon re-entry of the cells into GCs in an activation-induced deaminase (AID)-dependent process (90, 100). Earlier studies using a less complex (4‐hydroxy‐3‐nitrophenyl)acetyl (NP) antigen suggested that in the secondary responses, high affinity MBC-Ms matured and were able to become ASCs after booster immunization (107). More recent findings suggest that highly mutated and high affinity MBCs-M do not differentiate into ASCs, a process that is left for low affinity MBC-M in the primary immune response (92). It is likely that high affinity MBC-Ms secreting high affinity IgM have an important role in inflammatory and autoimmune disease such as rheumatoid arthritis (discussed in section 5.1) (108). It is speculated that high affinity MBCs-M class switch to other isotypes, as seen in tissue resident Fc Receptor Like 4 (FcRL4^+^) fractions in secondary lymphoid organs (SLO) and IgA plasma cells in the gut associated lymphoid tissue (GALT) (88, 92, 100). However, a recent study contradicted this notion and suggested that MBCs are unlikely to re-enter GCs in secondary responses for further diversification (102). It may be reasonable to speculate that the low affinity MBCs-M re-enter GCs for further mutation acquisition, to become high affinity MBCs-M with those that fail to do so becoming ASCs, whereas high affinity MBCs-M either contribute to the memory pool or class switch outside GCs as suggested recently (109). Whether high antigen valency, a feature of pentameric IgM, is a major contributing factor in decision making between high affinity MBCs-M and low affinity MBC-Ms is a fascinating area of research that needs further exploration (110).

In addition to MBCs-M, fate mapping studies using AID have also identified other subsets of MBCs-M in the spleen that spontaneously develop under germ-free conditions and are not derived from BM or gut (56). These MBCs-M display a lower mutation load compared to their class switch recombination (CSR) counterparts, suggesting residual antigen activation in the gut, from potential endogenous or food antigen (56). Additionally, they display large cross-reactivity, particularly against conserved N-glycans of bacteria and retroviruses (56). These MBCs-M display unmutated V_H_ genes with antibacterial activity, suggesting a pre-programmed antibody immune repertoire (56).

Box 1 **Key differences in effector B cell subsets.**

a. Short-lived plasma cells (SLPCs)—SLPCs of IgM producing antibodies are typically found in the spleen on the periphery of B cell follicles, displaying little to no SHM. SLPCs differentiation is governed by the BCL6 and BLIMP-1 transcription factor. SLPC release the early wave of antibodies post-antigen exposure and provide initial protective response prior to emergence of high affinity antibodies.

b. Long lived-plasma cells (LLPCs)—LLPCs continuously secrete antibodies at a constant titre. LLPCs also appear to be more stringently selected and appear in late GCs. LLPCs reside in BM, spleen, and gut-associated lymphoid tissues (GALTs).

c. Memory B cells IgM (MBCs-M)—MBCs secrete antibodies in response to cognate antigen challenge. MBCs maintain a higher diversity and appear much earlier in GCs. MBCs can be tissue resident or are found recirculating secondary lymphoid organs. MBCs-M display a lower mutation load compared to their CSR counterparts. MBCs-M display large cross-reactivity, particularly against conserved N-glycans of bacteria and retroviruses.

In humans, unswitched IgM memory B cells exist and are more abundant in local tissues such as GALT, lung, and SLOs compared to mice (88). MBCs-M have also been found in blood circulation (identified as IgM^+^IgD^+^CD27^+^) and show clonal relatedness to gut specific MBCs-M, IgM only PCs, and IgA only PCs (91, 111). Human gut IgM responses may involve IgM diversification from pre-existing IgM^+^IgD^−^CD27^+^ memory specificities, rather than *de novo* recruitment of naive IgM^+^IgD^+^CD27^−^ B cells, ensuring considerably faster CSR and providing protection to blood borne infections, possibly through cross-reactivity (91, 111). A recent study, which reported severe infections of *Klebsiella* in immunocompromised patients showed that these patients harbored *Klebsiella* LPS-O3 antigen specific MBCs in peripheral blood which showed clonal relatedness with intestinal plasmablasts (112). These MBCs were mostly MBCs-M, however, MBCs-G and MBCs-A were also found in circulation and closely related to IgA found in the lamina propria. Both MBCs-G and MBCs-A showed higher mutation rates (between 20 and 25 bp/IgHV gene) in their heavy chain variable regions, whereas MBCs-M showed less mutations (around 10bp/IgHV gene) in their VH (112). These antibodies were glycan-specific and bound to O3 antigen of the mannose residues present at the surface of other microorganisms, such as *Saccharomyces cerevisiae*, HIV and several other Gram^+^ and Gram^−^ human commensals (112). This is consistent with other studies showing human MBCs-M secreted IgM targeting mucus-embedded SIgA coated commensals in the ileum, thus assisting in providing protection from diverse bacteria (88). These MBCs-M are not limited to bacterial species and have been found in the blood of healthy adults mildly infected with human BK polyomaviruses (113). In such settings, MBCs-M were shown to have high viral neutralizing abilities against BK virus and were also pan-reactive against another related JC virus, which causes progressive multifocal leukoencephalopathy in immunocompromised individuals (113). Interestingly, these MBCs-M were functionally distinct from MBCs-G, lost their neutralizing functionality when Cμ was replaced by Cγ and were resistant to class switching to IgG producing cells (113).

MBCs are different to LLPCs in several ways—LLPCs continuously secrete antibodies at a constant titer, while MBCs only do so in response to cognate antigen challenge (101). Additionally, LLPCs reside in BM, spleen, and gut-associated lymphoid tissues (GALTs), whereas MBCs can be tissue resident or are found recirculating secondary lymphoid organs (SLOs) (94). LLPCs also appear to be more stringently selected and emerge in late GCs, whereas MBCs maintain a higher diversity and appear much earlier in GCs (103, 114). The higher diversity of MBCs provides an evolutionary advantage to the host where there is increased antibody breadth protection, a phenomenon that is critical in most antibody-based vaccine designs.

## Immunoglobulin M in Diseases

### Immunoglobulin M in Non-Communicable Diseases

An additional aspect where natural and antigen-induced IgM are thought to play non-redundant roles are autoimmune diseases and cancer. In autoimmune diseases such as systemic lupus erythematosus (SLE) and rheumatoid arthritis (RA), IgM and IgG titers are increased and associated with disease pathogenesis. In SLE, IgG autoantibodies directed against double stranded DNA (dsDNA) are thought to be pathogenic, while IgMs anti-dsDNA are thought to be protective (115). SLE patients are typically treated with B-cell depletion therapy, rituximab, with adverse outcomes of hypogammaglobulinemia linked to increased infections in these patients (116). In two studies using SLE prone mouse strains (MRL-lpr/lpr) and NZB x NZW that spontaneously develop SLE (characterized by severe immune complex-mediated glomerulonephritis and death by 12 months of age from renal failure), secreted IgM (sIgM) was shown to be essential in preventing disease (**Table 1**) (137, 138). When lpr mice were crossed with sIgM-deficient mice, they developed a severe form of the disease with increased glomerular immunocomplex deposition and IgG ds-DNA autoantibodies, which was rescued by treatment with IgM autoantibodies (138). In the second study, treatment of NZB x NZW mice IgM anti-dsDNA improved disease symptoms including reduction in renal pathology and organ damage (137).

**Table 1:** Role of immunoglobulin M (IgM) in infectious and non-infectious diseases.

Organism (disease)	Species	Function	Reference
Plasmodium (malaria)	*P. falciparum*	Anti-α-gal IgM antibodies protective in adolescence	(117)
	*P. chabaudi*	Anti-α-gal IgM antibodies protective when transferred to mice	(117)
	*P. berghei*	MBCs-M secrete high affinity IgM in GCs	(118)
Trypanosomes (trypanosomiasis)	*T. brucei brucei*	nIgM not protective, sIgM-deficient mice not susceptible	(119)
	*T. congolense*	nIgM not protective, sIgM-deficient mice not susceptible	(119)
	*T. evansi*	nIgM important for primary and secondary responses	(120)
Fungi (mycosis)	*C. neoformans*	nIgM and antigen IgM protects against systemic dissemination. Important for IFN-γ response and activation of macrophages.	(121, 122)
	*P. carinii*	nIgM protects against dissemination and priming of TH2 and TH17 responses	(123)
	*A. fumigatus*	Anti-GlcNAc IgM antibodies protect against allergic asthma	(124, 125),
Viruses (viral infections)	*Influenza A*	sIgM-deficient mice show poor viral neutralizing ability and increased viral titers	(126–128),
	*VSV*	Natural IgM traps VSV antigens in secondary lymphoid tissues	(129–131),
	*RVS*	IgM BCR on Bregs a target for RVS and detrimental to disease	(132)
Bacteria	*S. pneumonia*	Adoptive transfer of B1a cells derived sIgM led to improved survival of infected μMT mice. sIgM was dependent on GM-CSF	(133)
	*E. coli*	Adoptive transfer of B1a cells derived sIgM led to improved survival of infected μMT mice. sIgM was dependent on GM-CSF	(133)
	*Ehrlichia muris*	Bone marrow derived IgM-secreting cells, AID independent provide protection	(32, 134),
	*F. tularensis*	sIgM was directed at the LPS fraction of *F. tularensis* and depended on IL-1β	(135)
	*Haemophilus influenzae*	PD-L2 dependent B 1 natural IgM anti-phosphorycholine provide protection against H. influenzae	(136)
Non-infectious agents	SLE	Autoantibodies IgM anti-dsDNA are protective, sIgM mice protected	(115, 137, 138),
	Allergy	Anti-GlcNAc IgM antibodies passively administered or vaccine induced protective	(23, 124),
	Cancer	Natural IgM recognized sugar moieties include MUCIN 1, SAM6, PAM-1 in cancerous cells	

SLE, systemic lupus erythematosus; VSV, vascular stomatis virus; nIgM, natural IgM; ABPA, allergic bronchopulmonary aspergillosis.

In cancer, natural IgMs are associated with recognition and removal of precancerous cells, owing to their ability to recognize self-antigens of carbohydrate patterns and quickly activate the complement (7). The presence of natural IgM against specific sugar moieties not found in non-cancerous cells is also used as a diagnostic and a prognosis marker, particularly for breast cancers (**Figure 3**) (139). Some of these recognized sugar moieties include MUCIN 1 (140), SAM6/GPR78 (141), and PAM-1 (142), and have been proving to be useful as prophylactic and therapeutic targets when derived directly from a patient’s tumor cells (141, 142).

Very little is known about the role of natural and induced IgM in asthma, despite overrepresentation of asthma in patients with selective IgM syndrome (143, 144). Previous studies have suggested that neonatal vaccination with bacterial species, such as group A streptococcus containing GlcNAc or β-1,3-glucans can protect adult mice against *Aspergillus fumigatus* induced allergic asthma (**Table 1**) (10, 23, 124). Passive immunity with anti-GlcNAc natural IgM antibodies in adult mice protects against developing asthma, suggesting that these conserved germline-encoded IgM antibodies can have broad protective effects against other common allergens containing GlcNAc moieties, such as dermatophytes (124). B1 cells secreting IgM are also known to be stimulated by IL-5 and proliferate in an IL-33 receptor dependent manner (145). In this setting, IgM producing B1 cells promote oxazolone induced contact dermatitis in mice (145). Currently, it is unclear whether natural or secreted IgM plays different roles compared to membrane bound IgM, which is more likely to undergo class switching to IgE. More studies are needed to decipher the function of IgM in asthma beyond class switching.

### Immunoglobulin M in Shaping Mucosa Tolerance and Against Bacterial Infections

Microbiota colonize the mucosal sites soon after birth in humans and play key roles in homeostasis (146). The dominant antibodies found at mucosal sites are secretory IgAs, which binds and shapes microbiota (147–149). The majority of IgA plasma cells are generated from memory IgA B cells that reside in the lamina propria (LP) in the gut (150). In addition to IgA, emerging evidence places secreted IgM as a key player in maintaining local homeostasis at mucosal sites, such as the gut and lung, and assists in shaping local microbiota (9, 88). Here, we briefly discuss how local secreted IgM produced by memory IgM B cells shapes microbiota (as discussed under antigen-specific IgM memory cells). However, we mainly focus on discussing IgM contribution in regulating bacterial infections particularly in mucosal sites in experimental infection models (88). In the human gut mucosa, several studies have found human secreted IgM, together with secreted IgA, to coat human microbiota (88, 151, 152). IgM enhanced IgA binding repertoire and in some instances was even more potent in neutralizing enteric bacteria on its own (151). Specifically, IgM was found to promote bacterial species that are beneficial for healthy gut homeostasis, such as Firmicutes (e.g., *Bacillus cereus*, *Lachnospiraceae* spp. and *Ruthenibacterium* spp.) and Bacteroidetes (*Bacteroides vulgatus*) which are all beneficial (88, 146, 153). Age negatively correlated with the presence of these bacteria, resulting in dysbiosis in the adult population (153). Secreted IgM/MCBs-M may have developed to aid IgA in preserving microbiota homeostasis by directly interacting with bacteria to promote abundance of healthy microbiota and possibly eliminating pathogenic bacteria.

In the lung mucosa, infection of B cell deficient mice (μMT mice) with *Escherichia coli* or *Streptococcus pneumoniae* led to increased mortality and lung bacterial burdens (**Table 1**) (133). Transfer of wild type mice pleural cavity B1a cells, which secrete copious amounts of sIgM led to improved survival of infected μMT mice (133). Granulocyte-macrophage colony stimulating factor (GM-CSF) was found to be essential in sIgM B1a induced protection, as transfer of B1 cells lacking this cytokine did not rescue infected μMT mice (133). Induced sIgM produced by B1a cells has also been shown to be essential in *Francisella tularensis* infection (135). In this infection model, production of sIgM was directed at the LPS fraction of *F. tularensis* and depended on IL-1β for its earlier protective effects. Interestingly, sIgM showed great specificity to *F. tularensis* and did not cross-react with *E. coli* LPS, suggesting that it was induced sIgM, and not natural occurring sIgM (135).

Emerging evidence suggests a localized B cell repertoire in the lamina propria which can influence BM and peritoneal cavity B cell populations (9). Mono-colonization of germ-free mice influenced VDJ recombination process in the LP (9). In another study, neonatal immunization with group A streptococcus antigen increased GlcNAc reactive B cells and clonotype diversity in adult mice (10). These GlcNAc reactive B cells were educated in the LP in early life and disseminated systemically to provide protection against GlcNAc containing species (10). Early education of B cells might support diversification of the B cell repertoire but needs further investigations.

### Immunoglobulin M Against Fungi

Natural IgM antibodies directed against fungal pathogens are important in both complement-dependent and -independent fungal recognition and clearance (154) and have been shown to have direct killing effects (155). Most natural IgM antibodies are conserved across species and are not dependent on antigen exposure, as suggested from their presence in germ-free mice and umbilical cord blood of non-human primates and humans (123). In fungi, these natural IgM antibodies are directed to conserved major cell wall components β-(1,3)-glucan and chitin and are derived from B1 cells in the mouse spleen (**Table 1**) (121, 123, 154, 156).

Mice deficient of sIgM show increased dissemination of *Cryptococcus neoformans* to other organs such as spleen, kidney, and brain when infected intravenously (121). In this setting, sIgM is thought to contribute to the optimal Th1 induction and the subsequent activation of phagocytic macrophages that kill the fungus (121). B cells, and more specifically IgM, were shown to be essential in protective mechanisms against *C. neoformans* when naïve B cells were transferred to RAG-1-deficient mice (121, 156). Transfer of B cells was shown to reduce fungal dissemination to the brain but had no effect in lung fungal burden (156). Both natural and infection induced-IgM were important in the control of *C. neoformans* and contributed to the optimal Th1 cytokine production (121, 156). A human study using antibodies generated against *C. neoformans* glucuronoxylomannan in a transgenic mouse expressing human IgM, revealed that protective effects of IgM were epitope specific and route of injection dependent (122). Non-protective effects of sIgM have been observed when sIgM-deficient mice were injected intraperitoneally, with increase in their survival compared to control wild type mice (157). In Pneumocystis, an opportunistic fungi that infects HIV/AIDS patients, natural IgM antibodies are detected and have an important role in clearance (158). Mice lacking sIgM are susceptible to pulmonary *Pneumocystis carinii* infection and show increased burdens, which are associated with altered inflammatory response (**Table 1**) (123). Secreted IgM deficiency in mice is associated with reduced IL-6 and IL-1β innate cytokine production and adaptive TH2 and TH17 responses at both lung and draining lymph nodes (123). The susceptibility of sIgM-deficient mice to *P. carinii* infection is likely to be due to defective DC presentation and priming of CD4 T cells and a lack of class switching to protective mucosal IgG and IgA isotypes (123). Individuals with X-linked hyper-IgM syndrome due to CD40L mutation, display equal susceptibility to pulmonary fungal infections, which may suggest a minimal role for antibodies in these infections (159, 160). In both experimental models and in humans where sIgM or B cell antibody function was blocked by anti-CD20 monoclonal antibodies, severe defects in optimal innate and adaptive responses occurred, resulting in susceptibility to fungal infections. This is suggestive of a critical function of natural IgM.

### Immunoglobulin M Against Parasites

The role of antibodies in trypanosoma parasitic control are well documented, where a constant battle to opsonize and kill parasites occurs, while parasites have developed complex variant specific surface glycoproteins (VSGs) to avoid host recognition (**Figure 3**) (161, 162). *Trypanosoma evansi* can infect all domesticated animals and is transmitted by biting sand flies and vampire bats (163). Antibodies, particularly IgM isotype have been shown to be important in the control of *T. evansi* (120). Type 1 cytokines and effector molecules such as IFN-γ, TNF-α, and iNOS were found to be redundant in a mouse model of *T. evansi* infection. In contrast, mice deficient of IgM or B cells succumbed significantly quicker to *T. evansi* infection and were not able to control parasitemia (**Table 1**) (120). Furthermore, IgM, rather than IgG, was found to be critical in parasitemia control as passive transfusion with *T. evansi* immune IgM serum, but not IgG serum protected naïve mice from re-infection with the same parasite (120). Complement, which kills parasites through phagocytosis *via* complement receptor mediated recognition, did not play a role in this instance, suggesting other mechanisms of parasite killing. In a pleomorphic *Trypanosoma brucei* AnTat 1.1E infection model, B cells and IgM were found to play minimal roles in trypanosomiasis associated anemia, parasite induced anti-VSG antibodies, host survival, and disease progression (119). Mice lacking IgM showed similar levels of parasitemia to wild type counterparts when infected intraperitoneally, exposed to tsetse fly bites or non-virulent field isolates (119). Similarly, to *T. evansi* infections, in IgM-deficient infected mice, an increase in VSG specific-IgD isotype antibody production was observed, as well as normal levels of VSG specific-IgG2a or IgG3, which are thought to have compensated for the loss of IgM (129). Interestingly, B cells which are thought to induce immune pressure in pleomorphic *T. brucei* were found to be redundant in this instance and VSG intergenic switching occurred independently of antibody or IgM presence (119). A recent study showed an important function of natural and induced IgM antibodies against trypanosome lytic factors (TLF2) in *T. brucei* infected people (162). Healthy people were found to harbor germline encoded natural IgM antibodies against TLF2, which were further upregulated by *T. brucei* *rhodesiense* infection and reduced by treatment with suramin or melarsoprol (162). TLF2-IgMs antibodies interact with the TLF protein, haptoglobin related protein (HPR), thus offering a route for parasite endocytosis and killing *via* alternative complement activation (162).

IgM antibodies specific to α-gal have been shown to be protective against *Plasmodium falciparum*, a malaria causing parasite (117). IgM antibodies against α-gal are thought to be generated in the gut by microbiota that express α-gal, such as certain strains of *E. coli* (O86:B7) (23). In human, anti-α-gal IgM antibodies can directly bind to *P. falciparum* sporozoite and initiate complement activation and parasite clearance (117). Children between 0 and 1 years old in malaria endemic areas are at the highest risk of developing the disease, which is associated with reduced anti-α-gal IgM antibodies in serum. In older children the level of anti-α-gal IgM antibodies increases, associated with added protection from malaria parasite and this is partly attributed to the maturity of the B cell compartment. These anti-α-gal IgM antibodies were induced in germ-free animals mono-colonized with *E. coli* (O86:B7) strain and were found to be protective when these mice were infected with different malaria parasites (117). Interestingly, these anti-α-gal IgM antibodies did not depend on AID, suggesting that these were natural IgM antibodies generated outside germinal centers and did not undergo somatic hypermutation (117). Other natural IgM memory B cells able to recognize merozoite surface protein 1 (MSP1) protein of *P. falciparum* have been shown to be considerably more rapid than IgG and confer protection against re-challenge with the parasite (164). Similarly, to anti-α-gal IgM antibodies, these anti-MSP IgM B cells gave rise to mainly T cell-independent high affinity plasma cells (B220^+^CD138^+^) and T cell-dependent (B220^-^CD138^+^) IgM plasma cells (164). These IgM memory B cells produce IgM plasma cells with similar binding affinity to class switched IgG plasma cells (164). It is plausible to assume that these memory IgM B cells developed as a strategy to protect against primary and secondary Plasmodium infection to prevent dissemination of parasites pre-GCs B cells, capable of generating high affinity IgG plasma cells.

### Immunoglobulin M Against Viruses

Early control of viral infections is dependent on innate natural antibodies and most vaccine strategies target potent neutralizing antibodies. Natural IgM antibodies can bind to surface glycoproteins of most viral capsids and activate the complement system *via* classical pathways, leading to viral opsonization and killing (126). Influenza virus is a rapidly replicating respiratory virus that is detected by natural IgM antibodies, which do not require AID or class switch recombination or somatic hypermutated B cells (93). In the absence of adaptive immune cells, including B and T cells, such as in the case of severe combined immunodeficiency (SCID), influenza virus is uncontrollable and causes death in animals (127, 165). Mice lacking sIgM are susceptible to influenza virus and show poor viral neutralizing ability leading to increased viral titers (128). Adoptively transfer of naïve or influenza primed serum to sIgM-deficient or RAG-1-deficient mice restores viral neutralizing ability and virus clearance (**Table 1**) (128). Vesicular stomatitis virus (VSV), an enveloped RNA virus requires both natural IgM and complement for clearance (130). Human sera lacking any of the early complement factors C1−C5, but not late complement factors C6–C9 is unable to kill VSV infected cells. These complement factors rely on natural IgM presence on sera for effective killing of VSV infected cells (130). Interestingly, mice contain natural VSV IgM antibodies that were induced independently of infection (131). These antibodies were essential in limiting early VSV dissemination to vital organs, such as the kidney, brain, and lungs and neutralized the virus in secondary lymphoid tissues (131). In IgM-deficient mice or B cell-deficient mice, VSV was recruited to secondary lymphoid tissue, where it accumulated and activated the natural IgM antibody response (**Table 1**). This, in turn, delayed dissemination of VSV to the kidneys and brain and allowed activation of the adaptive immune response, thus reducing VSV titers at early time points in these tissues (131). Furthermore, IgM-deficient mice show a delayed antibody class switching to neutralizing IgG, which illuminated this trapping of VSV antigens in secondary lymphoid tissues by natural IgM (129, 131).

## Concluding Remarks

Classic memory takes up to 4 days to develop and may be slow relative to the rapid invasion of encapsulated bacteria and viruses. It is during this period that innate-like B cells, which produce rapid cross-reactive natural IgM or long-lasting antigen-specific IgM responses that can interfere with initial infection. As cross-reactive SLPC, they can assist phagocytes and complement, to clear the system and mucosal sites. Antigen-specific LLPC can rapidly class switch to specific isotype or become highly specific IgM producing cells able to clear infection or activate other adaptive cells. However, despite all this knowledge, little attention has been paid to their role in immune responses or how their production can be manipulated to the host’s advantage. The higher diversity of MBCs provides an evolutionary advantage to the host, where there is increased antibody breadth protection, a phenomenon that is critical in most antibody-based vaccine designs. We do not fully understand the role of IgM in allergies beyond class switching and its role in lung mucosal sites where it has been suggested that it can be hijacked by viruses to gain entry in mucosal sites. Whether natural or induced IgM can be fine-tuned to fight cancers and other infections is an area still less explored.

## Author Contributions

SH conceived the idea. KJ, AS, FB, and SH wrote the paper. All authors contributed to the article and approved the submitted version.

## Funding

This work was supported by ICGEB, Cape Town Component, Medical Research Council (MRC) South Africa as well as support by the South African National Research Foundation (NRF) Research Chair initiative (SARChi) and Wellcome Trust CIDRI-Africa (203135Z/16/Z) to FB. SH is supported by NRF Thuthuka Grant (117721), MRC Self-initiated grant.

## Conflict of Interest

The authors declare that the research was conducted in the absence of any commercial or financial relationships that could be construed as a potential conflict of interest.
